# Comparison of Body Composition, Muscle Strength and Cardiometabolic Profile in Children with Prader-Willi Syndrome and Non-Alcoholic Fatty Liver Disease: A Pilot Study

**DOI:** 10.3390/ijms232315115

**Published:** 2022-12-01

**Authors:** Diana R. Mager, Krista MacDonald, Reena L. Duke, Hayford M. Avedzi, Edward C. Deehan, Jason Yap, Kerry Siminoski, Andrea M. Haqq

**Affiliations:** 1 Department of Agricultural Food and Nutritional Science, University of Alberta, Edmonton, AB T6G 2R3, Canada; 2 Department of Pediatrics, University of Alberta, Edmonton, AB T6G 2R3, Canada; 3 Department of Paediatrics, University of Melbourne, Melbourne, VIC 3010, Australia; 4 Faculty of Medicine and Dentistry, Radiology, University of Alberta, Edmonton, AB T6G 2R3, Canada

**Keywords:** body composition, muscle strength, non-alcoholic fatty liver disease, Prader-Willi syndrome, children

## Abstract

Syndromic and non-syndromic obesity conditions in children, such as Prader-Willi syndrome (PWS) and non-alcoholic fatty liver disease (NAFLD), both lower quality of life and increase risk for chronic health complications, which further increase health service utilization and cost. In a pilot observational study, we compared body composition and muscle strength in children aged 7–18 years with either PWS (*n* = 9), NAFLD (*n* = 14), or healthy controls (*n* = 16). Anthropometric and body composition measures (e.g., body weight, circumferences, skinfolds, total/segmental composition, and somatotype), handgrip strength, six minute-walk-test (6MWT), physical activity, and markers of liver and cardiometabolic dysfunction (e.g., ALT, AST, blood pressure, glucose, insulin, and lipid profile) were measured using standard procedures and validated tools. Genotyping was determined for children with PWS. Children with PWS had reduced lean body mass (total/lower limb mass), lower handgrip strength, 6MWT and increased sedentary activity compared to healthy children or those with NAFLD (*p* < 0.05). Children with PWS, including those of normal body weight, had somatotypes consistent with relative increased adiposity (endomorphic) and reduced skeletal muscle robustness (mesomorphic) when compared to healthy children and those with NAFLD. Somatotype characterizations were independent of serum markers of cardiometabolic dysregulation but were associated with increased prevalence of abnormal systolic and diastolic blood pressure Z-scores (*p* < 0.05). Reduced lean body mass and endomorphic somatotypes were associated with lower muscle strength/functionality and sedentary lifestyles, particularly in children with PWS. These findings are relevant as early detection of deficits in muscle strength and functionality can ensure effective targeted treatments that optimize physical activity and prevent complications into adulthood.

## 1. Introduction

Obesity is defined as excessive adiposity, with a body mass index (BMI) above 30 kg/m^2^, that increases potential health risks for the individual [1]. More than 340 million children 5–19 years worldwide had obesity in 2016, reflecting almost a fivefold increase from 1975 [1]. Factors such as high birth weight, parental overweight and obesity, and parental education and income may increase risk for childhood obesity [2,3]. Rare genetic conditions, such as Prader-Willi syndrome (PWS), predispose children to increased adiposity, which may lead to obesity when triggered by environmental factors [3,4,5,6]. In contrast, the childhood obesity epidemic has been attributed mostly to unhealthy diets high in saturated fat and simple sugars and living environments that promote sedentary lifestyles and positive energy balance [7]. The increasing prevalence of obesity among children poses a significant public health challenge as it increases risk for adverse cardiometabolic disorders such as non-alcoholic fatty liver disease (NAFLD) and early onset insulin resistance [8,9,10,11], reduced quality of life, and higher mortality [12,13,14]. Childhood obesity increases long-term health service utilization and expenditure, placing significant burdens on healthcare systems and governments worldwide [15,16]. Active living is an integral part of current strategies for managing obesity including healthy eating, pharmacotherapy, and bariatric surgery in selected cases [1,17,18,19]. Yet, limited evidence suggests that certain obesity conditions, such as PWS, may cause reductions in lean body mass relative to overall fat mass [20,21]. This may undermine obesity management efforts by limiting participation in physical activity and related lifestyle modification.

PWS is the most common syndromic obesity condition, which stems from cytogenetic mutation at chromosome 15q11.2-q13 [22,23,24]. It is characterized by relative increased subcutaneous adiposity, lower visceral adiposity, reduced lean body mass, decreased muscle tone (hypotonia), lower basal metabolic rate and daily energy expenditure, and poor motor proficiency that limits physical activity in the face of hyperphagia [5,6,23,25,26,27,28,29]. PWS occurs in 1 in 100,000 to 300,000 children with differences in frequency and severity of impulse control, adaptive behavior, and intellectual ability depending on genotype [22,23,26]. Genotype is determined based on the primary mechanism causing cytogenetic mutation at chromosome 15q11.2-q13; deletion (approximately 75% of cases), uniparental disomy (UPD) (approximately 25% of cases), or imprinting defect (approximately 1–3% of cases) [22,23,24]. Deletion occurs via deletion of paternal genes from the 4–6 Mb region chromosome 15q11.2-q13 [22,23,24]. Imprinting defect is caused by the microdeletion of the imprinting center of chromosome 15q11.2-q13 [22,23,24]. UPD occurs via inheritance of two copies of a genetic locus from only one parent [30] and may present with higher IQ and less behavior problems, but higher rates of psychosis and co-occurring autism spectrum disorder compared to the deletion genotype [31,32,33].

NAFLD, on the other hand, is a common condition of non-syndromic obesity in which excess fat builds up in the liver and is characterized by total and central obesity with hyperinsulinemia, insulin resistance, altered liver function, and cardiometabolic dysregulation [34,35,36,37]. While underlining obesity may not have a specific genetic cause, several genes, including Phospholipase Domain-containing 3 (PNPLA3), are associated with NAFLD pathogenesis and affect predisposition to, progression, and severity of NAFLD by facilitating hepatic adipose accumulation which impacts lipid and glucose metabolism, resulting in liver damage [38,39,40]. Furthermore, microRNAs (miRNA) involved in lipid metabolism regulation, including miR-122, miR-192 and miR-375 [41], may predispose individuals to developing or progressing NAFLD due to gene expression alteration caused by environmental factors [41,42]. Prevalence of NAFLD in children is estimated at 13% (9.8% adjusted) and has increased significantly in the last decade given the worsening obesity epidemic [43,44]. NAFLD occurs in 1 in 3 male children and 1 in 4 female children with overweight and obesity [45,46].

PWS and NAFLD represent models of obesity but vary in genetic influence, body composition and cardiometabolic health, which may impact muscle strength, muscle functionality, and associated physical activity levels [47,48,49], further complicating identification and management strategies. Compared to the central adiposity characterized in NAFLD, those with PWS are known to have relative increased subcutaneous adiposity but lower visceral adiposity and lean body mass [23,25,26,27,47,48,49] and better metabolic profiles with increased insulin sensitivity [27]. Therefore, NAFLD is model of obesity with metabolic dysfunction whereas PWS is a model of obesity with relatively functional metabolism. However, no published studies have directly compared these two disorders to understand their etiologic differences. This pilot study was thus designed to examine associations between measures of body composition and cardiometabolic dysfunction with muscle strength and functionality in children with PWS and NAFLD to provide insight that informs the development of targeted management strategies for these contrasting models of obesity.

## 2. Results

### 2.1. Demography, Anthropometry, and Body Composition

Demographic and anthropometric data are summarized in Table 1. Twelve healthy controls and 2 children with PWS had body weights within healthy reference ranges. None of the children with NAFLD had body weights within normal reference ranges. Waist circumferences (WC) were within healthy reference ranges for 15 healthy controls, 6 PWS, and 4 NAFLD children (*p* < 0.001). Children with PWS have significantly less skeletal muscle mass, absolute and Z-scores, respectively, compared to children with NAFLD (11.5 ± 4.7 and −1.7 ± 0.9 vs. 20.9 ± 7.2 and 0.9 ± 0.9) (*p* > 0.01).

**Figure 1:**
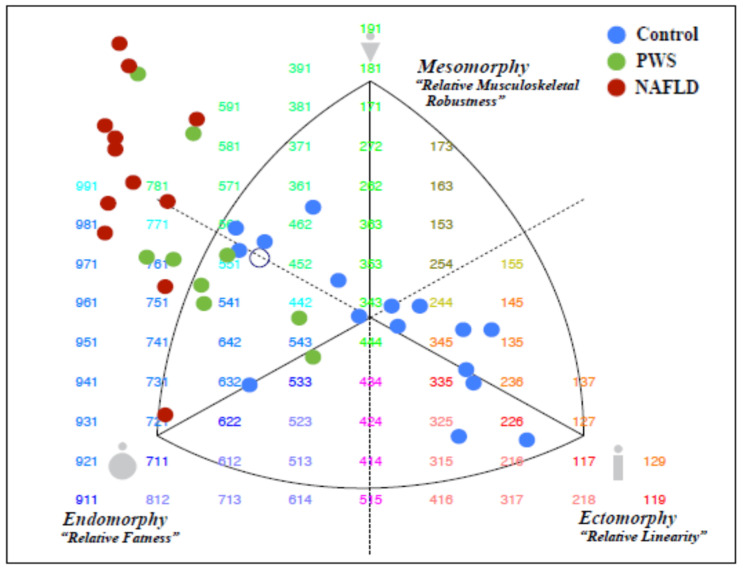
Somatoplot of Children with PWS, NAFLD, and the Healthy Controls. Ten different anthropometric measurements (height, weight, two circumferences [flexed arm and calf]), two bone breadths (humerus and femur), and four skinfolds (triceps, subscapular, supraspinal and medial calf) were entered into the Somatotype 1.2.5 software. The magnitude of the endomorphy, mesomorphy, and ectomorphy were plotted for healthy controls (*n* = 16), PWS (*n* = 9) and NAFLD (*n* = 12). Abbreviations: PWS, Prader-Willi syndrome; NAFLD, non-alcoholic fatty liver disease.

Somatotype data is summarized in Table 2 and Figure 1. All 16 healthy controls, 5 PWS, and 1 NAFLD had somatotypes within normal healthy reference ranges. Healthy controls had a mixed distribution of endo-ecto-meso-morphic body habitus, and all had somatotypes within healthy reference ranges. Healthy controls had significantly lower endomorphic habitus compared to PWS and NAFLD (*p* < 0.001). Both NAFLD and PWS had meso-endomorph body habitus; but NAFLD more so than PWS (*p* < 0.001). There was a significant difference between mesomorph habitus in healthy controls and NAFLD (*p* < 0.001) and between endomorph habitus in children and PWS and NAFLD (*p* < 0.001). Five PWS children and 1 NAFLD had somatotypes within normal healthy reference ranges. Children with PWS had relative overall adiposity, and significantly lower indices of lean body mass, particularly in the lower extremities (Table 3).

There were no significant associations between anthropometrics (weight, weight-z, height, height-z, BMI, BMI-z, WC, WC-z, skinfold measures/circumferences), somatotype characterization, or gender (*p* > 0.05). No other associations were observed between total/segmental (absolute or percent) measures of body composition.

### 2.2. Markers of Cardiometabolic Dysregulation and Liver Dysfunction

Serum alanine aminotransferase (ALT) concentrations above 20 U/L were observed in two children with PWS and all children with NAFLD (*p* = 0.02) (Table 4). Hyperinsulinemia (>20 mU/L) was observed in 2 PWS vs. 11 NAFLD children, while homeostasis model assessment for insulin resistance (HOMA-IR > 3) was observed in 3 PWS vs. 12 NAFLD children. One healthy control (age ~ 14.5 years) had both elevated serum insulin and HOMA-IR, likely secondary to pubertal development. Elevated serum fasting triglycerides (TG) levels were observed in 5 NAFLD children, 2 PWS children, and 1 healthy control (*p* < 0.05).

Compared to children with serum ALT < 20 U/L, those with serum ALT >20 U/L had significantly higher HOMA-IR (6.7 ± 1.7 vs. 1.9 ± 1.1; *p* < 0.001), TG (1.5 ± 1.0 vs. 0.9 ± 0.5 mmol/L; *p* = 0.02) and lower high density lipoprotein cholesterol (HDL-C) (1.1 ± 0.3 vs. 1.4 ± 0.3 mmol/L; *p* = 0.005).

### 2.3. Measures of Handgrip Strength, Six Minute-Walk-Test, Blood Pressure, Muscle Quality and Habitual Physical Activity

**Figure 2:**
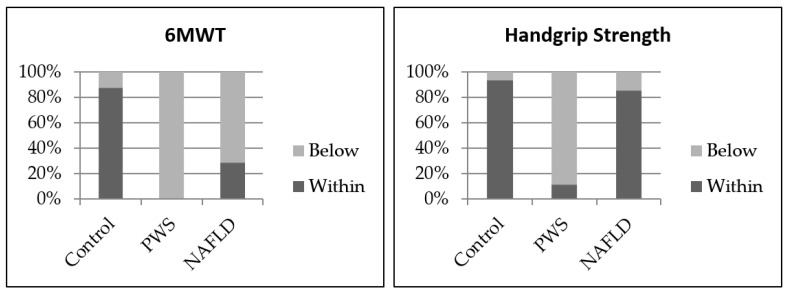
6MWT and Handgrip Strength Normative Values for Age and Gender. 6MWT distances were compared to reference values. Average handgrip strength scores of three trials for each hand were compared to Jamar^®^ Hydraulic Hand Dynamometer normative values. Scores < 2 standard deviations of the average value for age and gender were considered abnormal. Healthy controls (*n* = 16), PWS (*n* = 9) and NAFLD (*n* = 12). Abbreviations: 6MWT, six minute-walk-tests; PWS, Prader-Willi syndrome; NAFLD, non-alcoholic fatty liver disease.

Resting systolic blood pressure (BP) was significantly higher in the NAFLD group (126 ± 8 mmHg) relative to the PWS (116 ± 16 mmHg) and HC (115 ± 9 mmHg) groups (*p* = 0.01). Handgrip strength and 6-minute walk test (6MWT) results are presented in Figure 2. Children with PWS had significantly lower handgrip strength and 6MWT distances than children with NAFLD or healthy controls (*p* < 0.05). Muscle quality in children with PWS, 3.4 (3.1, 3.6), was significantly lower than those with NAFLD, 4.4 (3.0, 5.8) (*p* = 0.05) (Table 1).

**Figure 3:**
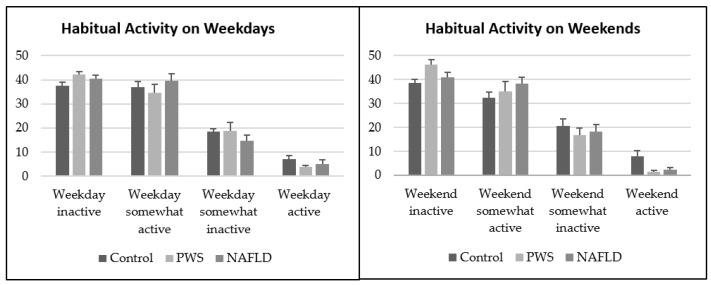
Percentage of Habitual Physical Activity on Weekdays and Weekends. Results are presented as percentage of hours for each day spent at four different activity levels: inactive (lying down), somewhat inactive (sitting), somewhat active (walking) and active (running). Healthy controls (*n* = 16), PWS (*n* = 9) and NAFLD (*n* = 12). Abbreviations: PWS, Prader-Willi syndrome; NAFLD, non-alcoholic fatty liver disease.

Physical activity is shown in Figure 3. Mean time spent in sedentary activities was 8.5 ± 0.68 (HC), 8.4 ± 1.9 (PWS), 7.9 ± 1.1 (NAFLD) hours/day (*p* > 0.05). Eight healthy controls and 3 children with NAFLD met current physical activity guidelines (60 min/day) for participation in active physical activity (*p* < 0.05). None of the children with PWS met current guidelines for time spent in active physical activity [50]. In addition, children with PWS had higher values for percentage of weekdays spent inactive (*p* = 0.02) and percentage of weekend days spent inactive (*p* = 0.007) compared to controls. Control children had a higher percentage of Saturday spent active compared to children with PWS (*p* = 0.04).

### 2.4. Associations between Anthropometric, Body Composition, and CardioMetabolic Markers

No significant associations were observed between somatotype phenotypes and serum markers of cardiometabolic dysregulation (*p* > 0.05). Body composition was not associated with time spent being active (*p* > 0.05). Endomorphic predominant phenotypes were associated with abnormal diastolic BP and systolic BP Z-scores (*p* < 0.05). Higher trunk-to-limb ratio was associated with serum TG > 1.5 mmol/L and HOMA-IR > 3 (*p* = 0.05). Increased android/gynoid ratios was associated with serum ALT > 20 U/L (*p* = 0.03). Significant inverse associations were observed between lean indices (appendicular-height, lean-height and lean body mass index) and time spent during the week on sedentary activities (*p* < 0.05).

Children with low 6MWT distance had higher insulin (26.2 ± 22.3 vs. 10.5 ± 8.9; *p* = 0.002), HOMA IR (6.1 ± 6.5 vs. 2.4 ± 2.1; *p* = 0.004) and diastolic BP (73.7 ± 8.7 mmHg vs. 67.9 ± 7.8 m; *p* = 0.04). However, children with both handgrip strength and 6MWT results outside of the reference ranges for age and gender had lower indices of lean body mass, lean height (absolute/Z-scores), skeletal muscle mass (absolute/Z-scores) and higher fat mass index (FMI) (*p* < 0.05). They also had increased serum concentrations of insulin (>20 mU/L), HOMA-IR >3 and ALT > 20 U/L (*p* < 0.05).

### 2.5. Associations between Genotype and Outcome Measures

DNA polymorphism analysis determined that 5 participants had UPD; fluorescence in situ hybridization (FISH) analysis determined that 3 participants had deletion genotype; and 1 genotype remained unknown. No additional testing, such as imprinting center microdeletion analysis [22], was requested to identify the unknown genotype due to the small sample size for this group. Diastolic BP Z-scores were significantly different between deletion and UPD genotypes, (1.78 (0.76–2.7) vs. 0.82 (0.38–1.5); *p* = 0.04). However, findings show that PWS children with deletion vs. UPD were older, 15.8 ± 2.7 vs. 10.7 ± 2.3, respectively, with expected differences in absolute anthropometric measurements due to age. There were no significant differences in sex, weight Z-score, height Z-score, WC Z-score, waist-to-hip ratio, systolic BP Z-score, and all laboratory measures between genotypes.

## 3. Discussion

We examined associations between body composition, muscle strength and physical activity in children with syndromic obesity with relatively functional metabolism (PWS), children with non-syndromic obesity with metabolic dysfunction (NAFLD), and healthy children (controls). Children with PWS had significantly lower measures of lean body mass and skeletal muscle mass compared to those with NAFLD. Reductions in lean body mass and skeletal muscle mass in children with PWS were associated with significant impairments in upper and lower muscle strength measures (i.e., handgrip, 6MWT tests) and reduced muscle quality, which translated to more time spent in sedentary activity when compared to NAFLD and healthy children. Notwithstanding, children with PWS had a better metabolic profile, including increased insulin sensitivity compared to the children with NAFLD [27], even though they had higher adiposity. Our study corroborates previous findings of characteristic visceral adiposity, low muscle mass and healthy metabolism observed in PWS [5,6,23,25,26,27,28,29] and the centralized adiposity, robust musculature, and metabolic dysfunction typical of NAFLD compared to healthy children [34,35,36,37].

Children with PWS primarily exhibited endomorphic phenotypes that indicate relative overall body fatness even when body weights were within normal reference ranges. Findings of predominantly endomorphic phenotypes, which is associated with large fat deposits and rounded (pear) body shape among children with PWS in this study, is consistent with the characteristic atypical body composition and fatness patterns featuring reduced lean tissue and increased adiposity in PWS [51]. The characteristic reduction in lean body mass, strength, flexibility, overall balance, and poor motor efficiency in the lower limbs has also been associated with reduced exercise capacity and participation and related high levels of sedentary behaviors [28,29]. Growth hormone therapy has been used to induce significant improvements in muscle size and lean body mass after two years in adults with PWS [52], with similar results reported in children [53]. However, our findings suggest that even after ongoing growth hormone therapy for >6 months, participants with PWS presented with significant impairments in muscle strength/functionality. Previous findings have shown that while growth hormone therapy enhances muscle thickness, both muscle growth and training effects (including weight bearing activities such as walking) were the more significant factors influencing overall muscle growth and motor development in infants and toddlers with PWS [54]. Furthermore, excessive adiposity may cause lipid infiltration in the muscle leading to decreased muscle quality and function [55,56,57]. Children with PWS may require targeted obesity management interventions including physical activity focused on muscle training to modify the characteristic body composition associated with the PWS to improve their quality of life.

Children with NAFLD, comparatively, displayed predominantly mesomorphic phenotypes which may explain the higher muscle strength observed among this group, enhancing their ability to meet physical activity guidelines when compared to children with PWS. Predominantly mesomorphic phenotypes are characterized by relative musculoskeletal robustness, little subcutaneous fat, broad shoulders and chest, small abdomen, and muscular and strong limbs. However, most of the children with NAFLD in our study had body weights outside normal references ranges with increased trunk/limb ratios and associated central adiposity, a phenotype typical of NAFLD [58]. The increased android/gynoid ratios found in children with NAFLD may also be an indicator for insulin resistance [59], which was supported by our findings. Hyperinsulinemia and insulin resistance were observed in a higher proportion of children with NAFLD, as well as significantly higher serum ALT concentrations and resting systolic BP compared to children with PWS and healthy controls. These findings are consistent with established cardiometabolic dysregulation and liver dysfunction in children with NAFLD [60]. Obesity, a marker of adiposity, remains a key predictor of NAFLD in children. As such, changing the anthropometric dimensions such as weight via targeted interventions including diet and physical activity may improve liver dysfunction and systolic BP, which is a predictor of cardiometabolic health.

BMI is utilized in clinical settings as the primary screening tool for obesity risk but has significant limitations as it cannot measure body composition or account for racial and sex differences [61,62]. Dual-x ray absorptiometry (DXA), a direct measures of body composition, is a more accurate screening tool for obesity but is not practical for clinical use due to cost, accessibility, and restrictions of use on healthy children, limiting its ability as an early identification tool for obesity. Our findings support somatotyping as a potential screening tool for obesity in children or adolescents. While somatotyping has been widely used to characterize body morphology and composition in order to determine performance and success in sporting competitions [63,64,65,66], its application in non-athletes remains poorly studied. Anthropometric dimensions impact the ability to perform physical activity, which in turn influences body dimensions and composition. This, and the impact of diet on body size and composition, form the basis for the growing interest among physicians, nutritionists, and sports specialists to employ somatotyping as a viable strategy for evaluating changes to an individuals’ body composition and shape for improved recovery from health disorders, quality of life and enhancing sports performance [67,68,69,70]. Our findings for the predominant endomorphic habitus of PWS and mesomorphic habitus of NAFLD support somatotyping as potential screening tool for assessing the level and location of adiposity, musculoskeletal development, and corresponding physical activity levels and sedentariness. Given that the anthropometric dimensions used for categorizing somatotypes respond to changes in diet and physical activity [36,67,68,71], future investigations are needed to validate somatotyping as a quick, global measure of changes in body composition in response to lifestyle, (e.g., diet and physical activity) and pharmacological (e.g., growth hormone or anti-obesity therapy) interventions. These unique differences in body composition combined with the differences in muscle strength and physical performance have potential implications for rehabilitation and therapy in children with these conditions.

This is one of the first studies to compare PWS and NAFLD as contrasting models of obesity, including the objective measurement of body dimensions and biochemical tests that assess various cardiometabolic parameters. These variables helped to address biases associated with self-reports. We were able to recruit a relatively sizable cohort of children with PWS despite the rarity of this syndromic obesity condition. PWS is a rare genetic condition, which makes recruitment of a larger number of participants difficult. Therefore, the participants were not precisely matched for the case–control design. Additionally, phenotypic expression of PWS may vary depending on genotype but we may have failed to detect such differences due to a limited sample size. As such, our relatively small sample size limited sub-analysis of data to examine patterns. However, a post hoc power analysis revealed sufficient power to determine associations between body composition and measures of muscle strength/function (α = 0.05 and β = 0.8). A larger sample size would allow for better case–control matches and permit assessment of factors such as gut microbiome, which has shown alterations previously in children PWS and NAFLD [72,73]. Additionally, genomic and transcriptomic analyses may be conducted in future investigations to gain insights into the underlying mechanisms influencing obesity in NAFLD and PWS [74,75,76]. Clinical identification of validated genetic NAFLD risk variants [38,39,40] and associated miRNA [41,77,78] as well as identification and validation of currently unknown NAFLD-associated genetics may assist in developing a robust genetic profile to enhance our understanding of NAFLD pathogenesis, aid in identifying new therapeutic targets, and improve precision medicine. Previous findings suggest that there may be a distinct miRNA profile for PWS compared to general obesity [74,75,76]. Analysis of miRNA in PWS may elucidate genetic similarities and differences between PWS genotypes which would enhance our understanding of the mechanisms underlying PWS. Further investigations should be conducted on larger cohorts to unravel clinically relevant differences that may exist between and within groups to enhance the genetic, metabolic, and phenotypic profiles of these models of obesity to aid in early detection and development of targeted obesity management interventions.

## 4. Materials and Methods

### 4.1. Participants

Healthy controls (*n* = 18), with healthy lipid panels and body weights within normal reference ranges [1] were recruited from the community via recruitment posters that were posted on community bulletin boards and social media. The recruitment fliers were approved by the Human Research Ethics Board at the University of Alberta. Two healthy controls were excluded from data analysis due to abnormal fasting lipid panel. No significant differences in anthropometric and demographic features were observed between healthy controls (*n* = 16) included in the analysis and those who were excluded (*p* > 0.05).

Children, 7–17 years old, with clinically diagnosed NAFLD (*n* = 14) were prospectively recruited from the Liver Clinic at the Stollery Children’s Hospital, Alberta Health Services between 2015 to 2017. Children attending the Liver Clinics for echogenic hepatic ultrasounds also underwent comprehensive metabolic and serological workup. The diagnosis of NAFLD was made in children based on elevated liver enzymes (ALT; aspartate aminotransferase, AST), the presence of hyperinsulinemia and hyperlipidemia, evidence of steatosis on liver ultrasound, liver biopsy where clinically indicated, and exclusion of other known causes of steatosis such as metabolic inborn errors of metabolism, alpha 1-antitrypsin deficiency, Wilson’s disease, hepatitis B or C and autoimmune hepatitis [36,37].

Children, 7–17 years old, with a clinical diagnosis of PWS (*n* = 9) and receiving recombinant growth hormone therapy for >6 months were prospectively recruited from the Endocrine Clinic at the Stollery Children’s Hospital, Alberta Health Services between 2015 to 2017. DNA methylation polymerase chain reaction (PCR) analysis was used to confirm the clinical diagnosis of PWS by detecting abnormalities within the imprinting region of chromosome 15q11.2-q13 [22,23,24]. FISH testing was used to identify the deletion genotype by analyzing the number, size, and location of DNA segments at chromosome 15q11.2-q13 [22]. UPD was also identified via DNA polymorphism analysis of chromosome 15q11.2-q13 of the parents and affected child [22]. If indicated, imprinting defect may be confirmed via specialized testing.

Children (1) with a history of a known primary liver disease associated with steatohepatitis not related to NAFLD (e.g., metabolic inborn errors of metabolism, alpha 1-antitrypsin deficiency, Wilson’s disease, hepatitis B or C and autoimmune hepatitis); (2) with a known primary diagnosis of type 2 diabetes mellitus or those on insulin; (3) on medications known to cause hepatic steatosis (e.g., corticosteroids, statins) or (4) with a history of comorbid conditions including other liver disorders or gastrointestinal disorders such as celiac disease were excluded from the study. This study was approved by the Health Research Ethics Board at the University of Alberta and all participants provided written informed consent (parents) and assent (participants) prior to participating in this study (Pro00056649).

### 4.2. Anthropometric Measurements

Height and weight were measured to the nearest 0.1 cm and 0.1 kg, respectively. Weight, height, and BMI were converted into Z-scores/percentiles using the World Health Organization standards [50]. Waist circumference was measured following the World Health Organization criteria and converted into Z-scores/percentiles [79].

### 4.3. Body Composition

#### 4.3.1. Dual-Energy X-ray Absorptiometry (DXA)

Whole body composition (i.e., total, percent, and regional lean mass; fat mass; and total mass) were measured using a Hologic Densitometer (4500A or Discovery A with Apex System 2.4.2, Waltham, MA, USA). DXA was performed as part of routine clinical care during annual evaluations for children with PWS (*n* = 8) and NAFLD (*n* = 7). Due to University of Alberta ethical restrictions, DXA was not performed for healthy children. Skeletal Muscle Mass (SMM) Z-scores were calculated according to age-gender reference populations [80]. FMI and lean body mass index were calculated as total fat mass or lean mass divided by height^2^ (m^2^) and were compared to reference values for age and gender [81] (Table 3).

#### 4.3.2. Skinfold and Bone Breadth Tests

Calf circumferences, skinfolds and bone breadths were measured according to standard procedures [36]. Trunk-to-extremity ratio (TER) was used as a measure of relative subcutaneous adipose tissue distribution and was calculated using the following equation:


$$TER=\frac{\;\mathrm{Subscapular}\;\mathrm{Skinfold}\;+\;\mathrm{Supraspinal}\;\mathrm{Skinfold}\;+\;\mathrm{Iliac}\;\mathrm{Skinfold}\;+\;\mathrm{Abdominal}\;\mathrm{Skinfold}\;}{\;\mathrm{Bicep}\;\mathrm{Skinfold}\;+\;\mathrm{Tricep}\;\mathrm{Skinfold}\;+\;\mathrm{Calf}\;\mathrm{Skinfold}\;}$$

For this study, skinfolds were measured using a large skinfold caliper (Beta Technology, Santa Cruz, CA) while humerus and femur diameters were measured using a small bone caliper (Calibres Argentinos). All measurements were made by one investigator, (KM), according to the International Society for the Advancement of Kinanthropometry (ISAK) methodology. A technical error of <5% for skinfold and <2% for circumferences was accepted.

#### 4.3.3. Somatotyping

Somatotyping has been used to aid the qualitative description of the relative risk for cardiometabolic dysregulation in various populations [36,67,68,71]. Briefly, somatotypes (i.e., ectomorph, mesomorph, and endomorph) are physical characterization of body morphology and composition. Ectomorphs tend to have relatively linear or slender frame, with low levels of fat and muscular tissue, broad and drooping shoulders, long thin limbs, and narrow thorax and abdomen. Mesomorphs have relative musculoskeletal robustness, little subcutaneous fat, broad shoulders and chest, small abdomen, and muscular and strong limbs. Endomorphs have relative adiposity, with large fat deposits, rounded body (pear) shape, large abdomen, rounded shoulders and head [68]. For this study, somatotyping was completed using the Somatotype 1.2.5 software (Sweat Technologies, Australia) [36,71]. Using the Heath-Carter approach, the software uses anthropometric measurements to evaluate somatotype and presents the individual’s classification in the somatotype graph. These measurements include height, body mass, bi-epicondylar breadths of the humerus and femur, girths of the arm’s calf and bicep in both flexion and tension, and skin folds (i.e., triceps, biceps, subscapular, supraspinal, suprailiac, abdominal, and calf). Skinfolds were measured using a Lange skinfold caliper (Beta Technology, Santa Cruz, CA). Humerus and femur diameters were measured using a small bone caliper (Calibres Argentinos). These measures were conducted as part of the research protocol according to the ISAK methodology and were measured by 1 investigator (KM) certified in this methodology [36,67,68,71]. A technical error of <5% for skinfold and <2% for circumferences was accepted.

### 4.4. Surrogate Markers of Liver and Metabolic Disease

Metabolic markers, including TG, HDL-C, low density lipoprotein cholesterol (LDL-C), total cholesterol (TC), insulin, glucose, ALT, aspartate amino transferase (AST), gamma-glutamyl transferase (ϒGT), alkaline phosphatase (ALP), albumin, urate, and C-reactive protein (CRP), were analyzed at the Core Laboratory of Alberta Health Services using standard methods [20]. ALT values >20 U/L were considered abnormal [82]. The homeostasis model assessment for insulin resistance (HOMA-IR) was used as an index of insulin resistance [83] using the following equation:


$$HOMAIR=\frac{\;\mathrm{fasting}\;\mathrm{glucose}\;\left(\frac{\mathrm{mmol}}{L}\right)*\;\mathrm{fasting}\;\mathrm{insulin}\;\left(\frac{\mathrm{mU}}{L}\right)}{22.5}$$

### 4.5. Muscle Strength, Six Minute-Walk-Test, Blood Pressure, Muscle Quality and Habitual Physical Activity

Handgrip strength was assessed using a Jamar^®^ Hydraulic Hand Dynamometer (Patterson Medical, Mississauga, ON, Canada). Six-minute walk tests were performed using standard procedures and scores below two standard deviations (SD) for age and gender were considered abnormal [84,85]. BP and heart rate were measured immediately before (after 10 min rest) and after the 6MWT using an Adview^®^ 9000 modular diagnostic station (American Diagnostic Corporation, NY, USA). BP was converted to Z-scores/percentiles [86]. Muscle quality of the upper arms was expressed as the muscle strength (dominant arm)/total arm lean muscle [87]. Physical activity was assessed using the validated Habitual Activity Estimation Scale questionnaire and children were asked to report physical activity on two days and a weekend within two weeks of the study visit [88].

### 4.6. PWS Genotyping

Genotyping was confirmed for eight out of nine participants with PWS via FISH testing for the deletion genotype and DNA polymorphism analysis for UDP [22,23,24,30,89]. Briefly, genotype identification is based on the primary mechanism causing cytogenetic mutation at chromosome 15q11.2-q13; mainly deletion, UDP, or imprinting defect [22,23,24,30]. FISH testing may be used to identify the deletion genotype by analyzing the number, size, and location of DNA segments at chromosome 15q11.2-q13 [22]. UPD may be identified via DNA polymorphism analysis of chromosome 15q11.2-q13 for the parents and affected child [22]. Imprinting defect may be confirmed via specialized testing if required. Since only 1 genotype was not identified via FISH or DNA polymorphism analysis, imprinting defect was suspected but further testing was not requested due to the small sample size for that genotype group.

### 4.7. Statistical Analysis

Data analysis was completed using the SAS 9.0 statistical software (SAS, Version 9.4; SAS Institute Inc., Cary, NC, USA). Data was expressed as median (interquartile range) for most intra- and inter-group assessments. Mean (standard deviation) was expressed for parametric data assessing the DXA results and the cohort. Data was assessed for normality using the Shapiro–Wilks test. Non-parametric variables were log transformed. Between group (HC vs. PWS vs. NAFLD) were performed using analysis of variance (ANOVA). Primary outcome variables including Z-scores for body composition measures, BP, handgrip strength, and 6MWT were adjusted for age and gender. *T*-tests were performed to compare segmental total body composition measured by DXA in PWS and NAFLD only. Univariate analyses were performed to assess associations between each variable, except DXA, for each group (PWS, NAFLD, and HC) and for the entire cohort. Multivariate analyses were performed to assess associations between body compositions measures, anthropometric measures, muscle strength/functionality, and cardiometabolic markers for the entire cohort. Statistical significance was considered at *p* value <0.05.

## 5. Conclusions

In summary, children with PWS in this pilot study exhibited relatively healthy metabolic profiles with body compositions that typify relative body fatness and reduced skeletal muscle which manifests as hypotonia, characterized by decreased muscle strength, muscle quality and increased sedentary activity. Comparatively, children with NAFLD exhibited body compositions that were reflective of relative muscular robustness and central adiposity, body weights outside healthy reference ranges, and associated liver and cardiometabolic dysfunction, which manifests as hyperinsulinemia, insulin resistance, and an increased risk for developing comorbidities such as type 2 diabetes mellitus. Clinical assessment of the genetic, metabolic, and phenotypic profiles for children with PWS and NAFLD is critical in determining the underlying mechanisms contributing to obesity in order to develop targeted screening tools and obesity management interventions for these conditions. Somatotype characterization may be an effective non-invasive tool within the clinical setting for identifying children with excessive adiposity that are at risk for muscle strength deficits; it is easy to perform and can provide important information regarding body composition changes over development and throughout treatment. Future studies of larger cohorts including additional genetic and metabolomic analysis and gut microbial assessment, may aid in developing well-rounded, robust profiles of these models of obesity. This will help to improve detection and management of pediatric obesity and associated comorbidities. Early identification of children with overweight and obesity that may have potential cardiometabolic dysfunction and/or deficits in muscle strength and functionality is necessary to ensure effective rehabilitation strategies are developed to optimize physical activity in children based on their model of obesity.

## Figures and Tables

**Table 1:** Demographic, Anthropometric and Related Measurements.

	HC (*n* = 16) ^1^	PWS (*n* = 9) ^1^	NAFLD (*n* = 14) ^1^	HC vs. PWS *p* Value ^2^	HC vs. NAFLD *p* Value ^2^	PWS vs. NAFLD *p* Value ^2^
Gender (M:F)	9:7	2:7	8:6	NS	NS	NS
Age (years)	12.7	13.0	13.6	NS	NS	NS
	(10.8, 14.5)	(9.8, 15.1)	(11.6, 15.4)			
Weight (kg)	41.7	42.5	88.4	NS	<0.0001	0.002
	(37.3, 55.7)	(33.2, 63.2)	(67.8, 101.7)			
Height (cm)	154	143	162	NS	NS	0.007
	(144, 167)	(129, 154)	(151, 168)			
BMI (kg/m^2^)	17.9	21.3	32.8	0.02	<0.0001	0.007
	(16.8, 20.1)	(18.5, 28.1)	(27.8, 37.3)			
Weight	0.51	0.6	3	<0.0001	<0.0001	NS
Z-score ^3^	(−0.19, 0.87)	(−0.22, 1.5)	(2.2, 2.9)			
Height	0.48	−1.3	0.05	0.0007	NS	0.002
Z-score ^3^	(−0.11, 1.43)	(−1.9, −0.37)	(−0.13, 1.5)			
BMI	−0.1	1.2	2.9	0.004	<0.0001	0.0003
Z-score ^3^	(−0.79, 0.56)	(−0.67, 2.22)	(2.5, 2.9)			
Waist (cm)	65.7	75.5	95.7	0.04	<0.0001	0.003
	(62, 71.4)	(66.4, 87.6)	(88.9, 114.7)			
Waist	−0.3	0.8	1.8	0.005	<0.0001	0.0002
Z-score ^3^	(−0.52, 0.27)	(0.31, 1.3)	(1.5, 2.1)			
WHtR ^4^	0.42	0.5	0.6	<0.0001	<0.0001	0.04
	(0.41, 0.45)	(0.5, 0.6)	(0.5, 07)			
WHtR ^4^	−0.67	1.0	1.8	<0.0001	<0.0001	0.01
Z-score ^3^	(−1.0, −0.09)	(0.5, 1.6)	(1.4, 2.0)			
Systolic BP Z-score	0.78	1.2	1.6	NS	NS	0.006
	(0.019, 1.0)	(0.4, 2.5)	(1.2, 1.9)			
Diastolic BP Z-score	0.15	0.9	1.0	NS	0.01	NS
	(−0.2, 0.57)	(0.6, 1.6)	(0.7, 1.3)			
Handgrip Strength (kg)	18.3	9	23.7	0.002	0.001	NS
	(16.7, 29.2)	(3.9, 12.8)	(17.7, 32.2)			
6MWT (m)	603	436	489	NS	NS	NS
	(569, 634)	(379, 459)	(460, 522)			
Muscle Quality ^5^	n/a	3.4	4.4	n/a	n/a	0.05
		(3.1, 3.6)	(3.0, 5.8)			

^1^ Values are expressed as median (IQR). ^2^ *p* values <0.017 are considered statistically significant with correction (Bonferroni) for multiple pairwise corrections.^3^ Determined using World Health Organization anthropometric calculator (Canada, 2014 revision). ^4^ Waist-to-height ratio (WHtR) calculated as waist circumference (cm)/height (cm). ^5^ Muscle quality calculated as muscle strength (dominant arm)/total arm lean muscle. Abbreviations: BMI, body mass index; HC, healthy controls; Ht-to-Wt, height-to-weight ratio; NAFLD, non-alcoholic fatty liver disease; PWS, Prader-Willi syndrome; n/a: not available; NS, not significant.

**Table 2:** Somatotypes of Children with Prader-Willi Syndrome (PWS) and Non-Alcoholic Fatty Liver Disease (NAFLD) and Healthy Controls (HC).

	HC (*n* = 16) ^1^	PWS (*n* = 9) ^1^	NAFLD (*n* = 12) ^1^	HC vs. PWS *p* Value ^2^	HC vs. NAFLD *p* Value ^2^	PWS vs. NAFLD *p* Value ^2^
Endomorph	3.2	5.7	7.1	0.0085	<0.001	<0.001
	(2.8–4.1)	(5.0–6.2)	(6.4–7.4)			
Ectomorph	3.9	0.9	0.1	<0.001	<0.001	0.21
	(2.8–4.7)	(0.3–1.9)	(0.04–0.6)			
Mesomorph	3.5	4.7	7.6	0.002	<0.001	0.06
	(2.9–4.2)	(3.5–6.7)	(5.4–8.8)			

^1^ Values are expressed median (IQR). ^2^ *p*-value of <0.017 was considered significant; *p* values are corrected for post hoc pairwise comparisons using Bonferroni correction. Abbreviations: PWS, Prader-Willi syndrome; NAFLD, non-alcoholic fatty liver disease; HC, healthy controls.

**Table 3:** Body Composition in Children with PWS and NAFLD as measured by DXA.

Variable	PWS (*n* = 8) ^1^	NAFLD (*n* = 7) ^1^	*p* Value
Adipose Indices			
Fat mass total (kg)	22.9 ± 11.0	35.4 ± 14.4	NS
Fat mass/Height^2^ (kg/m^2^)	11.1 ± 4.2	13.7 ± 3.6	NS
Fat mass/Height^2^ Z-score	1.2 ± 0.6	1.7 ± 0.3	NS
Android/Gynoid ratio	0.9 ± 0.1	1.1 ± 0.1	0.006
Trunk/Limb fat mass ratio	0.8 ± 0.2	0.9 ± 0.2	NS
Trunk/Limb fat mass ratio z-score	0.5 ± 1.1	1.5 ± 0.6	NS
Fat Mass Index	10.8 ± 4.1	13.5 ± 3.4	NS
Lean Indices			
Lean mass total (kg)	27.3 ± 8.8	43.6 ± 13.0	0.01
Lean Mass/Height^2^ (kg/m^2^)	13.2 ± 2.4	17.2 ± 2.6	0.009
Lean Mass/Height z-score	−0.2 ± 0.9	1.2 ± 1.1	0.03
Lean Body Mass Index	12.9 ± 2.4	16.9 ± 2.5	0.008
Skeletal Muscle Mass (kg)	11.5 ± 4.7	20.9 ± 7.2	0.009
Skeletal Muscle Mass Z-score	−1.7 ± 0.9	0.9 ± 0.9	0.0001
Appendicular Lean/Height^2^ (kg/m^2^)	5.3 ± 1.2	7.6 ± 1.2	0.003
Appendicular Lean/Height^2^ Z-score	−0.7 ± 0.9	1.3 ± 0.5	0.0003
Lean Mass to Fat Mass Ratio			
Left Arm	1.0 ± 0.2	1.1 ± 0.2	NS
Right Arm	0.9 ± 0.2	1.0 ± 0.2	NS
Trunk	1.5 ± 0.5	1.4 ± 0.3	NS
Left Leg	1.0 ± 0.1	1.2 ± 0.1	0.01
Right Leg	0.9 ± 0.1	1.2 ± 0.1	0.001
Total	1.3 ± 0.2	1.3 ± 0.2	NS

^1^ Values are means ± SD (range). *T*-tests were used to compare differences in means with significance considered at *p* value < 0.05. Abbreviations: DXA, dual-x ray absorptiometry; NAFLD, non-alcoholic fatty liver disease; PWS, Prader-Willi syndrome; NS, not significant.

**Table 4:** Biochemical Measures of Liver and Cardiometabolic Dysfunction.

	HC (*n* = 16) ^1^	PWS (*n* = 9) ^1^	NAFLD (*n* = 14) ^1^	HC vs. PWS *p* Value ^2^	HC vs. NAFLD *p* Value ^2^	PWS vs. NAFLD *p* Value ^2^	Reference Values ^3^
ALT (U/L)	15	20	45	NS	<0.0001	0.0003	<20
	(14, 16.5)	(13, 28)	(37, 84)				
AST (U/L)	23	26	32	NS	0.001	NS	2–9 yrs: <50
	(21, 26)	(21, 33)	(27, 51)				≥10 yrs: <40
GGT (U/L)	5	5	7	NS	0.005	NS	Male: <70
	(5, 5)	(4, 9)	(4.9, 28)				Female: <55
ALP (U/L)	230	169	152	NS	NS	NS	5–17 yrs
	(181, 274)	(123, 223)	(117, 227)				100–500
Glucose (mmol/L)	5.1	4.9	4.9	NS	NS	NS	3.3–6.0
	(4.7, 5.2)	(4.7, 5.1)	(4.6, 5.4)				
Insulin (mU/L)	5.9	13.5	29	NS	<0.0001	0.009	5.0–20.0
	(4. 2, 9.4)	(9.9, 21.4)	(21, 50)				
HOMA-IR	1.2	3.0	5.9	NS	<0.0001	0.01	3.16
	(0.9, 2.1)	(2.1, 4.8)	(3.9, 12.8)				
TG (mmol/L)	0.7	1.1	1.4	NS	0.02	NS	<1.5
	(0.6, 1.0)	(0.6, 1.5)	(1.0, 2.3)				
TC (mmol/L)	3.9	4.2	4.4	NS	NS	NS	<4.4
	(3.5, 4.2)	(3.7, 5.3)	(3.7, 4.7)				
HDL-C (mmol/L)	1.4	1.2	1.1	NS	0.001	0.02	>1.0
	(1.3, 1.6)	(1.1, 1.7)	(0.9, 2.3)				
LDL-C (mmol/L)	2.0	2.4	2.5	NS	NS	NS	<2.8
	(1.8, 2.4)	(1.8, 3.4)	(2.1, 2.7)				
Albumin (g/L)	47	46	43	NS	0.02	NS	35–50
	(45, 48)	(43, 47)	(42, 46)				
Urate (umol/L)	244	310	346	NS	0.0003	NS	≤9 yrs: 100–300
							10–17 yrs: Male: 135–510 Female: 180–450
							≥18 yrs: Male: 180–500 Female: 150–400
	(211, 294)	(243, 365)	(321, 411)				
CRP (mg/L)	0.4	2.0	2.2	NS	<0.0001	NS	≤10
	(0.1, 0.7)	(0.6, 6.9)	(1.6, 3.9)				

^1^ Values are expressed median (IQR). ^2^ *p*-values < 0.016 was considered statistically significant to account for multiple pairwise comparisons. ^3^ Pediatric reference ranges obtained from Alberta Health Services: http://www.albertahealthservices.ca/assets/wf/lab/wf-lab-chemistry-reference-intervals.pdf (accessed 1 August 2022). There were missing values for urate in the control group (*n* = 1) and GGT in the NAFLD group (*n* = 1). Abbreviations: ALT, alanine aminotransferase; ALP, alkaline phosphatase; AST, aspartate transaminase; BP: blood pressure; CRP, C-reactive protein; GGT, gamma-glutamyl transferase; HC, healthy controls; HOMA-IR, homeostatic model assessment of insulin resistance; HDL-C, high density lipoprotein cholesterol; LDL-C, low density lipoprotein cholesterol; NAFLD, non-alcoholic fatty liver disease; PWS, Prader-Willi syndrome; TC, total cholesterol; TG, triglyceride.

## Data Availability

Not applicable.

## Abbreviations

6MWT	Six minute-walk-test
ALP	Alkaline phosphatase
ALT	Alanine aminotransferase
AST	Aspartate aminotransferase
BMI	Body mass index
BP	Blood pressure
CRP	C-reactive protein
DXA	Dual-energy x-ray absorptiometry
FISH	Fluorescence in situ hybridization
FMI	Fat mass index
ISAK	International Society for the Advancement of Kinanthropometry
ϒGT	Gamma-glutamyl transferase
HDL-C	High density lipoprotein cholesterol
HOMA-IR	Homeostasis model assessment for insulin resistance
LDL-C	Low density lipoprotein cholesterol
miRNA	microRNA
NAFLD	Non-alcoholic fatty liver disease
NS	Not significant
PCR	Polymerase chain reaction
PNPLA3	Patatin-like Phospholipase Domain-containing 3
PWS	Prader-Willi syndrome
SSM	Skeletal muscle mass
SD	Standard deviation
TC	Total cholesterol
TER	Trunk-to-extremity ratio
TG	Fasting triglycerides
UPD	Uniparental disomy
WC	Waist circumference
WHtR	Waist to height ratio
